# Thermostability of Organobentonite Modified with Poly(acrylic acid)

**DOI:** 10.3390/ma16103626

**Published:** 2023-05-09

**Authors:** Beata Grabowska, Sylwia Cukrowicz, Karolina Kaczmarska, Sylwia Żymankowska-Kumon, Artur Bobrowski, Bożena Tyliszczak, Natalia Maria Mrówka

**Affiliations:** 1 Faculty of Foundry Engineering, AGH—University of Krakow, Reymonta 23, 30059 Krakow, Poland; 2 Faculty of Materials Engineering and Physics, Department of Materials Engineering, Cracow University of Technology, 37 Jana Pawła II Av., 31864 Krakow, Poland; 3 Fundry Institute, Technische Universität Bergakademie Freiberg, 09599 Freiberg, Germany

**Keywords:** bentonite, poly(acrylic acid), organobentonite, composites, degradation, foundry

## Abstract

A new type of organobentonite foundry binder composed of a composite of bentonite (SN) and poly(acrylic acid) (PAA) was analyzed using thermal analysis (TG-DTG-DSC) and pyrolysis gas chromatography mass spectrometry (Py-GC/MS). The temperature range in which the composite retains its binding properties was identified using thermal analysis of the composite and its components. Results showed that the thermal decomposition process is complex and involves physicochemical transformations that are mainly reversible at temperatures in the ranges of 20–100 °C (related to evaporation of solvent water) and 100–230 °C (related to intermolecular dehydration). The decomposition of PAA chains occurs between 230 and 300 °C, while complete decomposition of PAA and formation of organic decomposition products takes place at 300–500 °C. Dehydroxylation of montmorillonite (MMT) in bentonite begins at about 500 °C, which leads to a drastic structural transformation. An endothermic effect associated with the remodeling of the mineral structure was observed on the DSC curve in the range of 500–750 °C. The produced SN/PAA composite was found to be thermostable during degradation in both oxidative and inert atmosphere, similar to the starting bentonite, and even maintained over a relatively higher and wider temperature range compared to organic binding materials used. At the given temperatures of 300 °C and 800 °C, only CO_2_ emissions occur from all the examined SN/PAA samples. There is no emission of compounds from the BTEX group. This means that the proposed binding material in the form of the MMT-PAA composite will not pose a threat to the environment and the workplace.

## 1. Introduction

**Figure 1:**
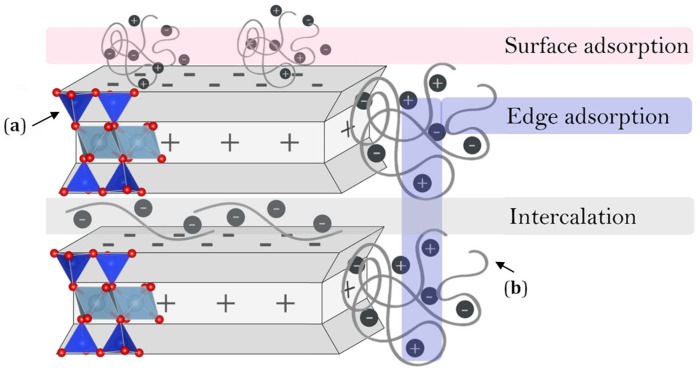
Scheme of some of the possible interactions of montmorillonite in calcium bentonite (**a**) with the chains of poly(acrylic acid) and (**b**) surface and edge adsorption combined with the intercalation [13].

The indication of new material solutions in foundry process engineering is expected by the metallurgical industry, due to the ecological problems still present in the production processes, as well as the progressive increase in the price of raw materials and energy. Inorganic and organic materials in bulk form are directed mainly to the preparation of molding compounds, which must meet technological, ecological and economic requirements. They must also be characterized by adequate thermal stability (thermostability) at high temperatures, which is one of the most important factors determining their suitability in technology, including foundry [1,2,3,4]. Synthetic molding compounds, which are mixtures of mineral matrix, bentonite, water and carbon additives, are widely used in industry. The mixture is molded, and a cavity of a specific shape of the target casting is shaped in the formed mold. The liquid metal is poured into the mold, usually a metal alloy, and after it solidifies, a casting of the predetermined shape is obtained. It is important to ensure that the high-temperature process produces a casting without surface defects. Hence, carbon additives in the form of carbon dust or synthetic resins are just introduced into the molding sand. These additives are carriers of lustrous carbon. When the liquid metal alloy is poured into the mold, they undergo thermal degradation with the formation of so-called pyrolytic carbon, which is a mixture of amorphous carbon and lustrous carbon (LC). In doing so, physical adsorption of LC on the surface of quartz matrix grains, also heated to a temperature in the range of 650–1200 °C and even 1400 °C, occurs. The lustrous carbon layer formed, of about 10^−1^ μm thickness, adhering to the matrix grains, provides a barrier against the penetration of liquid alloy, between the matrix grains, since, being non-wettable by liquid metal, it prevents its mechanical penetration deep into the molding sand and chemically isolates the mold material from the metal. Thus, it prevents chemical reactions between the oxides of metals and non-metals included in the alloy and the components of the molding sand, so that the surface of the casting is smooth and free from surface defects associated with burning of the molding sand. Carbon additives, therefore, on the one hand, are an indispensable ingredient for preventing casting defects [4,5], while on the other hand, they are a source of increased emissions of highly harmful volatile organic compounds (VOCs), polycyclic aromatic hydrocarbons (PAHs), benzene, toluene, ethylbenzene and xylenes (BTEX). When pouring liquid metal into a mold, gaseous decomposition products enter the atmosphere. They also remain in the composition of spent molding sand (i.e., left over from the metal pouring process), posing a hazard during their storage, and making them difficult to recycle [6,7]. Efforts are therefore being made to obtain effective and environmentally friendly replacements for commonly used carbon additives in synthetic molding compounds [8,9]. One possible solution is an organic structural modification of montmorillonite (MMT), the main component of bentonite. Such a route to obtain a new binder was undertaken by the team of authors of this article. The specific crystal structure and high surface chemical reactivity of MMT, as a hydrated layered aluminosilicate, makes it possible to change its properties through reactions with selected organic compounds, including intercalation (Figure 1). These reactions can occur through an interlayer exchange of cations with organic ions, adsorption of inert molecules, or a grafting process occurring between reactive surface groups of the mineral and organic molecules of the modifier [10,11,12]. Regardless of the method of interaction, the formation of montmorillonite intercalated with polymers offers the possibility of developing a new precursor of the desired carbon structure, i.e., lustrous carbon, as a result of thermal destruction of the organic part of the composite, without losing the binding properties of the casting binder. In addition, the proper matrix of the organic modifier is a key step in the mineral modification process, which determines the level of environmental safety of the new inorganic–organic binder. Due to the promising physicochemical properties questioning the possibility of generating harmful compounds during high-temperature pyrolysis, the team conducted MMT modification in calcium poly(acrylic acid) (PAA) bentonite [13]. PAA has reactive carboxyl groups (–COOH) in its structure. Full dissociation of these functional groups provides the macromolecule with a high negative charge density [14,15]. At the same time, the molecular weight distribution of acrylic polymers is wide, since they are synthesized industrially by radical polymerization of acrylic acid. In this application, it is important for PAA to have the lowest possible average molar mass to increase its ability to be intercalated between MMT packages. At the same time, poly(acrylic acid) is one of the most widely used water-soluble anionic polyelectrolytes, used in the production of hydrogels, superabsorbents and ion-exchange resins, as well as dispersing and binding agent [16,17]. In addition, it is used as a food additive due to its low toxicity [18]. Properties such as hydrophilicity, non-toxicity and binding capacity are valuable in terms of the requirements for casting binders. Therefore, the selected polymer is a promising material in terms of modifying the structure of the main component of bentonite. Ultimately, the modification was carried out using the interaction of active functional groups derived from combining inorganic (MMT) and organic (PAA) components to obtain an inorganic–organic composite. By FTIR spectroscopy and XRD analysis, it was confirmed that intercalation occurred, and BET confirmed polymer adsorption on the MMT surface. As a result, it was proven that there is partial intercalation of PAA into the inter-packet obsolescence of MMT, as well as the adsorption of the polymer on its surface [13].

This article completes a series of published papers related to the use of the obtained inorganic–organic composite (MMT-PAA composite) as a matrix grain-binding material, while serving as a precursor for lustrous carbon [13]. The authors present the results of its thermal analysis later in the article. Here, it should be noted that due to the action of high temperature pouring with liquid metallic alloy (about 1400 °C), degradation and destruction of the binder inside the mold occur with limited access to oxygen. The process of thermal degradation of the starting components of the obtained bentonite is known and described in the literature, both for poly(acrylic acid) and for bentonite [10,19,20,21,22]. However, in the case of the organobentonite considered in this article (in three component variants), there are no data in this regard. It should be noted that the course of binder degradation in the molding sand is therefore complex and occurs according to mixed degradation mechanisms (oxidative–non-oxidative degradation course) [23]. This is because in the composition of the binder, there are oxygen atoms (hydroxyl, carboxyl groups); hence, during the pouring of the liquid metal alloy into the mold, due to the strong stresses arising at the metal–form interface and the propagating heat wave, the course of oxygen degradation can also be expected. In addition, the cured mold is a heterogeneous system, in which there may be blocked air bubbles, so that the oxygen present may cause thermal degradation according to the mechanism of oxidative degradation. Hence, thermal analysis (TG-DSC) was carried out in both an inert and oxidative atmosphere of the composite, taking into account its starting components. The inclusion of the starting components of the composite in the thermal study, according to the authors, allowed a more accurate recognition of the course of their degradation process.

The introduction of a new binding material for casting production requires comprehensive knowledge of its binding properties, the technological properties of foundry masses, and the quality of the resulting castings with its use, as well as the course of its thermal degradation. The research results in this area conducted for the new binding material under consideration (organobentonite MMT-PAA) have already been largely published. They found that the produced organobentonite meets the requirements for binding materials, and the resulting casting has no surface defects [13,17,24]. The only remaining issue is to discuss the research topic related to its thermal stability. The thermoanalytical studies carried out in this article allowed for the examination of the course of thermal degradation, determination of thermal stability, and identification of the level of harmful gas emissions generated during casting production. The acquired knowledge is of significant importance for the transformation of materials in the form during the high-temperature process of pouring liquid metal alloy into the mold. It should be noted that it is not possible to determine in detail the mechanism of degradation of the bonding material under real conditions, i.e., during the process of pouring the liquid metal alloy into the mold. The mold is a complex system with local air gaps. In this case, thermal degradation of the bonding material will follow an oxygen mechanism. In contrast, in regions where such gaps are not present, degradation will occur without oxygen, hence the reaction mechanism will be different. The binding material should maintain its binding strength long enough to prevent liquid metal from penetrating deep into the mold and causing defects in the castings. It is important to know whether the modification of MMT will affect its thermal stability. In addition, the qualitative analysis of the decomposition products will allow for an assessment of the level of volatile organic compound emissions compared to bentonites already used in foundry, including those with carbon additives.

## 2. Materials and Methods

### 2.1. Materials

Thermoanalytical studies were conducted for the following:

1. Calcium bentonite (SN, ZGM Zębiec S.A., Zębiec, Poland). It is characterized by its cation exchange capacity determined by the Cu(II)-TET adsorption method: 65.3 meq·100 g^−1^ clay; MMT content: 69.2%. For the determination of CEC, water was introduced into the weighed amount of bentonite in a weight ratio of 1:20, and then the whole was dispersed with ultrasound. To the obtained suspension, 10 mL of 0.01 M Cu(II)-triethylenetetramine solution was added, after which the whole was centrifuged until a clear solution required for photometric measurement was formed. Spectrophotometric determination of the resulting solution was carried out at 620 nm in a 10 mm cuvette versus water as a blank. CEC was determined, taking into account the prepared calibration curve and the measurement difference. MMT content (%) was determined relative to a bentonite standard of known MMT content. The swelling index is 8 cm^3^·2 g^−1^. The chemical composition of the unmodified SN bentonite is as follows: SiO_2_ 67.39%, Al_2_O_3_ 18.96%, MgO 4.58%, CaO 3.02%, Fe_2_O_3_ 2.73%, Na_2_O 1.28%, K_2_O 1.13% (manufacturer’s data).
2. Poly(acrylic acid): average molar mass *M_w_* = 1800 g·mol^−1^ (PAA, Merck, Saint Louis, MA, USA).
3. Organobentonites SN/5PAA, SN/15PAA, SN/25PAA formed by the modification of calcium bentonite with poly(acrylic acid). The preparation of the modification is presented below in Section 2.2.

### 2.2. Preparation of Composite Materials

Organobentonite composites were prepared by modification of calcium bentonite with poly(acrylic acid) (PAA). Polymer solutions in concentrations of 5, 15 and 25% by weight of bentonite were added to mineral suspensions of 5 g calcium bentonite per 100 ml of distilled water that had been pre-dispersed in a laboratory stirrer (300 rpm, 3 h). Aqueous polymer solutions were prepared by dissolving the appropriate amount of PAA in 20 mL of distilled water. The mixtures were homogenized for 6 h in a laboratory stirrer at 300 rpm and then left to stand for 1 week modification process. The stirring operation was repeated, and the resulting dispersions were centrifuged (8000 rpm, 12 min). After separation from the unreacted polymer, each organobentonite precipitate was dried to a constant weight at 105 °C and then milled in an agate mortar. The first series of organobentonites were obtained: SN/5PAA, SN/15PAA and SN/25PAA [13,24].

### 2.3. Characterization Methods

Taking into account the results of structural studies and molecular simulations indicating a higher chemical affinity of poly(acrylic acid) to montmorillonite in calcium bentonite, thermal analysis was carried out for a series of organobentonites: SN/5PAA, SN/15PAA and SN/25PAA. The thermal studies were aimed at determining the thermostability of the produced modifiers to use them as binding materials in synthetic molding compound technology. In addition, qualitative analysis of the decomposition products of selected samples was carried out by the method of pyrolysis gas chromatography mass spectrometry.

#### 2.3.1. Thermogravimetry and Differential Scanning Calorimetry (TG-DSC)

Thermogravimetry and differential scanning calorimetry (TG-DSC) studies were performed using a NETZSCH STA 449 F3 Jupiter thermal analyzer that allows simultaneous TG and DSC measurements, thus providing comprehensive information on the thermal characteristics of the test sample. TG-DSC measurement parameters were as follows: temperature range: 25–800 °C; heating rate: 10 °C/min; atmosphere: oxidative (synthetic air) and inert (nitrogen, purity > 99%; Air Liquide, Krakow, Poland); gas flow rate: 40 mL/min, crucible material: platinum and sample mass: 10 mg. In addition, the DTG curve for the tested samples was also recorded.

#### 2.3.2. Pyrolysis Gas Chromatography Mass Spectrometry (Py-GC/MS)

The identification of gaseous products formed during thermal degradation of PAA and SN/PAA samples was carried out using the pyrolysis gas chromatography mass spectrometry method (Py-GC/MS).

In this method, the following instrumentation was used: the pyrolyzer “Py” Pyroprobe 5000 (CDS Analytical Inc., Oxford, PA, USA), the gas chromatograph “GC” Focus GC (Thermo Scientific, Waltham, MA, USA), coupled with the mass spectrometer “MS” Focus ISQ (Thermo Scientific). The study is based on transforming a solid sample (2–3 mg) into gas (also the so-called “fast pyrolysis”) by heating in an atmosphere of inert gas (helium) in a pyrolyzer, which is accompanied by thermal decomposition. The obtained mixture of compounds (pyrolysate) is separated on a chromatographic column in a chromatograph (RTX™-35 MS; capillary column; 30 m; ø0.25 μm, film thickness: 0.25 μm; stationary phase: 35% diphenyl/65% dimethyl polysiloxane; Restek™, Bellefonte, PA, USA). The gas chromatography “GC” conditions were as follows: an initial temperature of 40 °C (3 min hold) was raised at 3 °C/min to 100 °C (3 min hold) and then at 20 °C/min to 250 °C (3 min hold) using a constant helium flow of 1 cm^3^/min during the whole analysis. The temperature of the split injector was 250 °C and the split ratio was 1: 30. The transfer line temperature was 250 °C. The EI ion source temperature was kept at 250 °C. The ionization occurred with a kinetic energy of the impacting electrons of 70 eV. The gas products were identified based on the mass spectral library NIST MS Search 2.0 Libera (Chemm. SW, Version 2.0, Fairfield, CA, USA) using the Xcalibur program (ver. 2.2, Xcalibur, Arlington, VA, USA). Pyrolysis was carried out at 300 °C and 800 °C.

## 3. Results and Discussions

The results of TG-DTG-DSC thermal analysis are presented below, which were conducted to obtain knowledge about the course of thermal decomposition for the composite’s starting components, as well as organobentonite with varying polymer content. The obtained results will allow for the determination of the thermal stability of the new MMT-PAA binding material, which is important from the aspect of conducting the casting process with its participation. In addition, Py-GC/MS analysis and its results will expand knowledge about the emission of substances released into the atmosphere during the process of pouring liquid metal into the mold.

### 3.1. TG-DTG-DSC Studies

**Figure 2:**
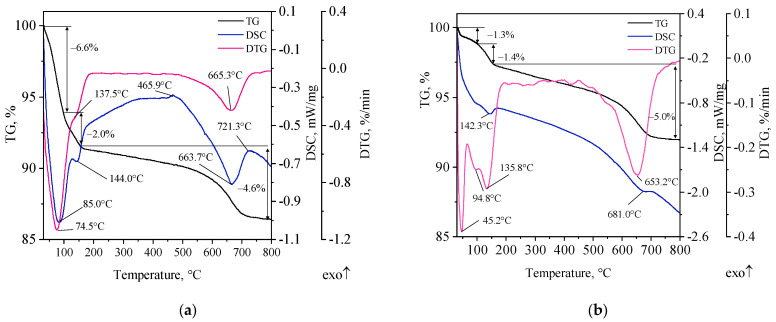
TG-DTG-DSC curves of calcium bentonite: (**a**) under an oxidative atmosphere, (**b**) under an inert atmosphere.

**Figure 3:**
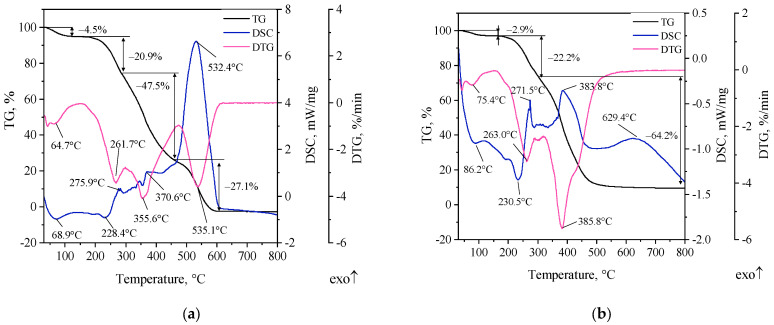
TG-DTG-DSC curves of poly(acrylic acid): (**a**) in an oxidative atmosphere, (**b**) in an inert atmosphere.

**Figure 4:**
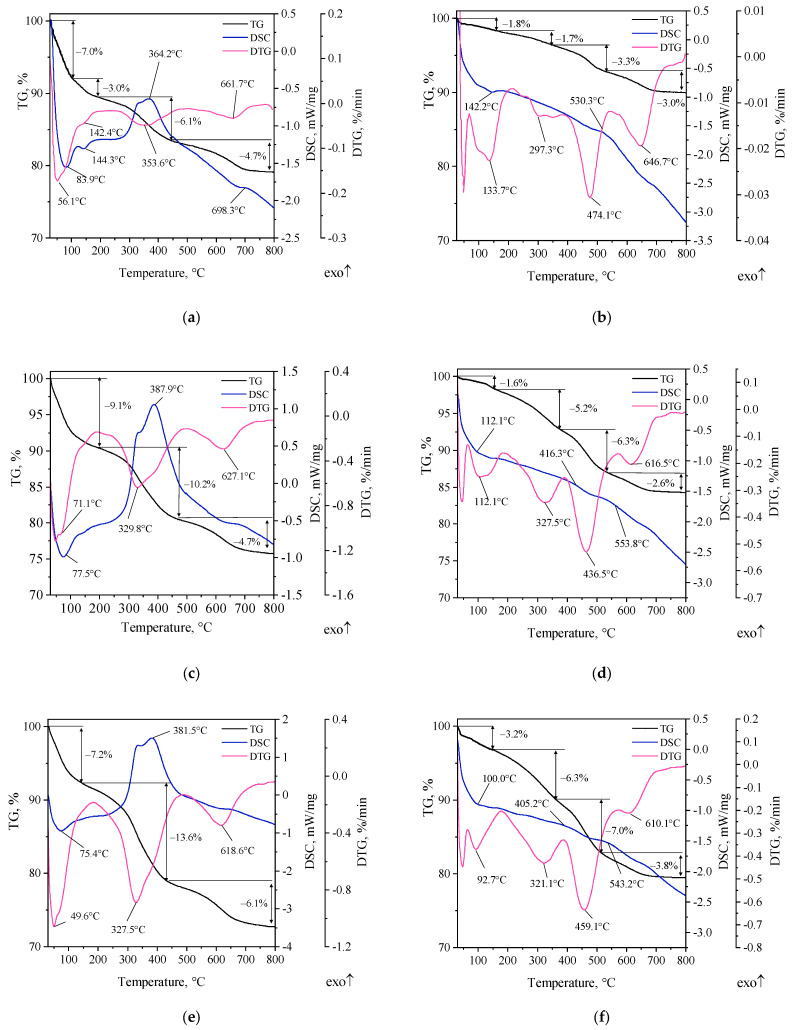
TG-DTG-DSC curves of organobentonites: (**a**) SN/5PAA under an oxidative atmosphere, (**b**) SN/5PAA under an inert atmosphere, (**c**) SN/15PAA under an oxidative atmosphere, (**d**) SN/15PAA under an inert atmosphere, (**e**) SN/25PAA under an oxidative atmosphere, (**f**) SN/25PAA under an inert atmosphere.

TG-DTG-DSC curves of the starting materials, i.e., calcium bentonite and poly(acrylic acid) (SN and PAA), and the group of modified bentonites are shown in Figure 2, Figure 3 and Figure 4. Due to the conditions in the casting mold during its pouring with liquid metal alloy, thermal tests were carried out in both oxidative (synthetic air, purity > 99.999%, Air Liquide; Krakow, Poland) and inert (nitrogen) atmosphere. It is considered that thermal degradation of the bonding materials in the casting mold occurs with oxygen access, and its mechanism is mixed, i.e., an oxidative–inert process [4,23]. Therefore, thermal analysis was conducted in the temperature range of 25–800 °C.

Three mass losses were observed on the TG curve of calcium bentonite: 6.6%, 2.0% and 4.6% (Figure 2a). Two of these mass changes in the temperature range of 100–150 °C correspond to dehydration of water adsorbed on the mineral surface and MMT inter-pack water, with the maximum mass loss rate seen on the DTG curve at 74.5 °C and 137.5 °C. An accurate indication of the temperature values at which the release of water weakly bound to the aluminosilicate structure (so-called hygroscopic water) and inter-pack water occurs is not possible [25,26,27]. These changes were accompanied by two endothermic effects (DSC curve). A third mass change with a maximum rate of loss at around 665 °C, associated with dehydroxylation of the mineral, begins at 500 °C. While dehydration does not change the basic structure of the T–O–T packages that build MMT, dehydroxylation is associated with a drastic structural transformation. This is because it leads to the reorganization of the octahedral layer resulting in the loss of the characteristic dioctahedral properties of smectites, including adhesion properties, ion exchange capacity and surface acidity. In the range of 500–750 °C, a clear endothermic effect related to the remodeling of the mineral structure was noted on the DSC curve [25,28]. The total mass loss after heating the calcium bentonite sample to 800 °C in an oxygen atmosphere was 13.2%. Thermal analysis of SN under an inert atmosphere showed a total mass loss of the material equal to 7.7% (Figure 2b). The course of the TG curve is clearly smoother. A change in the course of the DTG curve in the temperature range of 100–150 °C is also evident, suggesting a reverse order of dehydration of the mineral’s surface and inter-pack water, with the attribution of individual effects to the respective processes still unclear. Despite the lack of clear thermal changes in the DSC curve compared to the analysis in an oxygen atmosphere, two peaks are visible, corresponding to endothermic effects associated with the dehydration (142.3 °C) and dehydroxylation (681.0 °C) processes taking place.

Figure 3 shows TG-DTG-DSC curves of poly(acrylic acid) in oxidative and inert atmospheres.

Four PAA mass losses were recorded in an oxygen atmosphere (Figure 3a). The first is seen in the temperature range 30–200 °C, with a maximum decomposition rate at around 68 °C and a mass loss of 4.5%. The peak seen in the DTG curve at 64.7 °C corresponds to an endothermic effect at 68.9 °C attributed to reversible evaporation of the solvent and constitutional water. The second mass loss (20.9%) starts at ~170 °C and ends at 300 °C, with a maximum rate at 261.7 °C. In this temperature range, an endothermic effect was recorded at 230.4 °C, which corresponds to the formation of polymer anhydride (the process of desorption and elimination of water from carboxyl groups and the process within the same polymer chain). Above 300 °C, a dehydration reaction occurs with the formation of intermolecular bonds between –COOH groups of adjacent polymer chains (crosslinking) [29,30,31,32]. The next stage of PAA decomposition ends at ~475 °C, with a maximum rate of change at 355.6 °C and a mass loss close to half of the initial sample mass, i.e., 47.5%. The first exothermic effect observed in the DSC curve with a maximum at 370.6 °C is attributed to the decomposition of PAA anhydride chains [33]. Fragmentation of PAA chains can be linked to bond breaking in the –COOH group. The last polymer mass loss was recorded up to 615 °C with a maximum rate at 535.1 °C and a loss of 27.1%. Corresponding to the peak at 535.1 °C on the DTG curve, the strong exothermic peak at 532.4 °C on the DSC curve may indicate the final thermal degradation of PAA and the intense formation of gaseous destruction products in combustion reactions. Based on the analysis of TG-DTG-DSC curves, it can be concluded that the decomposition process of PAA in an oxygen atmosphere is completed at about 600 °C with a total mass loss of 100%. On the other hand, the total mass loss of the PAA sample in an oxygen-free atmosphere was 89.3% (Figure 3b). The residue (10.7%) most likely consisted of carbonized carbon, which is important with regard to assessing the possibility of lustrous carbon generation. The decomposition process of PAA in an inert atmosphere can be considered completed at a temperature of about 500 °C. The course of decomposition occurs with fewer thermal effects and intensity of mass loss. It occurs with three mass losses. The course of the TG curve in the polymer pyrolysis process is milder compared to the decomposition process in an oxygen atmosphere, although a more rapid but partial decomposition of PAA chains is noticeable (DTG curve, maximum mass loss rate at 385.8 °C). In addition to two endothermic effects (86.2 °C—surface-adsorbed water, less strongly bound; 230.5 °C—constitutional water, more strongly bound in the polymer structure) related to the loss of physically bound water, the DSC curve shows distinct three exothermic peaks (271.5 °C; 383.8 °C; 629.4 °C) corresponding to the thermal decomposition of the polymer at the respective temperatures and the release of gaseous destruction products in reactions occurring in an inert atmosphere.

Thermal studies of organobentonites were mainly concerned with establishing their thermostability, which is of key importance in the context of gaining more knowledge about the occurrence of possible phenomena during the process of pouring the mold with liquid metal in the considered system. TG-DTG-DSC curves of organobentonite are summarized in Figure 4.

TG curves recorded under an oxygen atmosphere for organobentonites showed three (SN/15PAA and SN/25PAA) to four (SN/5PAA) stages of mass loss, with the change in mass of modifiers under the given temperature regime increasing as the proportion of polymer in the material increased (Figure 4a,c,e). This is a direct consequence of the increased ratio of PAA to bentonite weight. The recorded mass losses with a maximum rate (DTG curves) in the temperature range of 30–200 °C indicate dehydration processes (solvent evaporation, dehydration) of both the mineral and PAA, which is also evidenced by endothermic effects visible in the DSC curves.

The onset of thermal degradation of the polymer in all considered cases of modifiers can be considered a temperature value of about 250 °C. The progressive degradation of PAA is expressed by clearly visible exothermic effects on the DSC curves of all organobentonites. The mass losses registered above 600 °C with the maximum rate of change at 661.7 °C, 646.7 °C and 618.6 °C for SN/5PAA, SN/15PAA and SN/25PAA, respectively, correspond to the values of the temperature of reorganization (dehydroxylation) of MMT, which has a destructive effect on the binding properties of bentonite. This shows the unfavorable effect of a higher proportion of the polymer (15 and 25% weight share) on the thermostability of the modifier, and further on the limitation of the applicability of the casting technology. On the other hand, the level of thermostability of the considered systems does not differ significantly from the thermostability of the starting bentonite, and even remains in a relatively higher temperature range compared to the organic binding materials used. The obtained level of thermostability of organobentonites makes it possible to carry out the process of flooding the mold with liquid metal and obtain a casting without surface defects associated with premature thermodestruction of the binding material (organobentonite) and undesirable reactions involving decomposition products at the metal–form interface.

The course of TG-DTG-DSC curves of organobentonites in an inert atmosphere is more complex (Figure 4b,d,f). Regardless of the proportion of polymer in the systems, a four-stage mass loss is observed in the temperature range of 30–800 °C. The main difference is an additional stage of mass loss with a maximum rate of change in the temperature range of 350–600 °C, which indicates the decomposition of chains formed by desorption and elimination of water from the carboxyl groups of PAA anhydride. Accordingly, the pyrolysis of organobentonites divides the decomposition of the polymer, also in the mineral structure, into two stages: the decomposition of polymer chains and the final thermal decomposition. Above 500 °C, the surface-adsorbed or inter-pack polymer chains have most likely charred (carbonized carbon), although quantitative analysis of its residue in the MMT structure is difficult due to the complexity of the system. Thermal effects are barely discernible. Noteworthy is the fact that the presence of residual carbon from PAA thermal decomposition is probable, and of technological importance in the context of the possibility of forming lustrous carbon in the destruction process. However, the thermal analysis carried out here does not provide complete information. The process of thermal decomposition of the considered organobentonites is very complex, and it is even more complicated under real conditions during mold pouring with liquid metal. Hence, obtaining full knowledge in this regard would require planning a separate cycle of both thermal and structural studies, which would be material for a separate scientific and research work. At the same time, it should be noted that both during degradation occurring in an oxidative and inert atmosphere, it is close to the thermostability of the initial bentonite, and even maintains a relatively higher and wider temperature range compared to the organic binding materials used [5,7,9,34,35].

The multi-stage course of structural changes in organobentonite was compared with the literature studies. It was confirmed that the introduction of an organic additive to bentonite is associated with a change in the course of mass changes to a more complex. Two DTG peaks between 200 and 600 °C were observed in organobentonites and they confirm the modification of bentonite with PAA, but these peaks are not observed in natural, unmodified bentonite SN [36,37,38].

Details of the information obtained from the analysis of TG-DSC curves of organobentonites are summarized in Table 1. The table also includes the results of the thermal analysis of unmodified SN bentonite.

### 3.2. Py–GC/MS Studies

**Figure 5:**
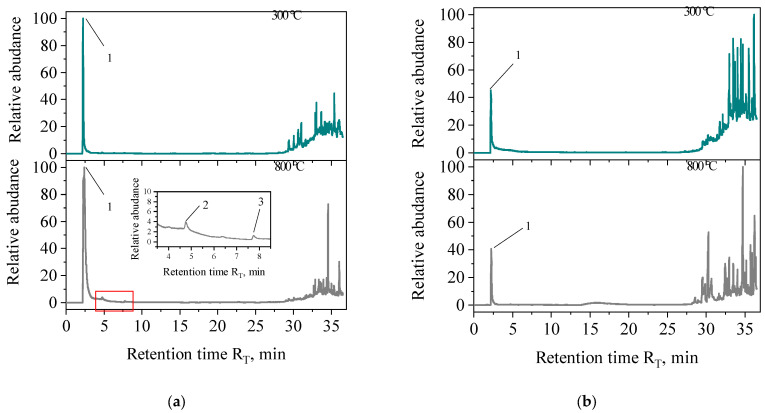
Py-GC/MS chromatograms of (**a**) PAA and (**b**) SN/PAA (representative example—SN/25PAA).

Qualitative analysis of decomposition products released at a selected temperature was performed for samples—PAA compared to SN/PAA samples—using the method of pyrolytic gas chromatography coupled with mass spectroscopy (Py–GC/MS). Measurements were conducted at 300 °C and 800 °C (the maximum temperature). The choice of the measurement temperature of 300 °C was associated with the fact that at this temperature, there is increased thermal decomposition. No studies were conducted for the MMT sample as it does not contain an organic fraction. The PY-GC/MS studies are qualitative in nature. The focus was on determining the emission of CO_2_ and volatile compounds from the BTEX group, as the determination of BTEX compounds is a standard for evaluating the quality and environmental protection aspects of the materials used in foundries. However, in publication [24], the authors’ team provides additional knowledge in this area. Figure 5 shows the Py–GC/MS results of the PAA and samples (for retention data and peak identification, see Table 2).

From Figure 5 it was found that at 300 °C, the decomposition of PAA sample took place with the formation of CO_2_ registered with the chosen measurement method (Table 2). The identification of CO_2_ (high peak) at 800 °C indicated the progressive process of decomposition. Aromatic hydrocarbons were formed by decomposition of the binder at 800 °C in the form of benzene and toluene (Table 2).

On the other hand, in the case of the examined samples of SN/PAA organobentonites, CO_2_ emissions were recorded. No emissions of compounds from the BTEX group were detected. This fact means that the introduced polymer as an organic component of the MMT-PAA composite will not have a negative impact on the environment and will not increase the carbon footprint.

## 4. Conclusions

During the pouring the liquid metal into the mold, thermal degradation of mold components of a mixed (oxidative and non-oxidative mechanism) nature occurs due to the resulting stresses at the metal–form interface and the propagating heat wave. This is because the cured mold containing the bonding material is a heterogeneous system, in which there may be blocked air bubbles, so that the oxygen present may become the cause of thermal degradation according to the mechanism of oxidative degradation. The course of thermal degradation of the binding material is complex and multistage, which is due to the structure, physicochemical properties of the considered organobentonites with different PAA content. At the same time, it depends on the conditions of the decomposition carried out (oxidative environment or oxygen-free environment); therefore, the mechanism of their decomposition can only be given with some probability.

Thermal analysis (TG-DTG-DSC) in both inert and oxidative atmospheres was carried out for organobentonite produced in three compositional variants (SN/5PAA, SN/15PAA and SN/25PAA), taking into account its starting components, to further determine its thermostability, as well as to discern the course of thermal decomposition. It was found that as the temperature increases, physicochemical transformations associated with the evaporation of solvent water (20–100 °C) take place. This is followed by the release of bound water (100–230 °C). Up to about 230 °C, mainly reversible processes take place. As the temperature increases, the decomposition of poly(acrylic acid) chains proceeds (230–300 °C). In the temperature range of 300–500 °C, PAA completely decomposes with the release of gaseous decomposition products from a group of organic compounds, including BTEX, as confirmed in the authors’ publication [24]. The part of the polymer that has not decomposed at about 550 °C may contain carbonized carbon, which is important for the use of organobentonite as a binder from generating lustrous carbon. The authors conducted technological tests of SN/PA organobentonite-bonded masses and confirmed the positive effect of PAA on casting quality. The made casting had a better surface quality than a casting made in the classical way, i.e., in a mold made of sodium bentonite-bonded molding sand [24]. It was found that the dehydroxylation of montmorillonite (MMT) in bentonite starts at a temperature of about 500 °C. Dehydration does not change the basic layered structure of the MMT, but dehydroxylation is associated with a drastic structural transformation. In the range of 500–750 °C, a clear endothermic effect associated with the remodeling of the mineral structure was noted on the DSC curve. It was confirmed that the thermostability of the produced SN/PAA composite, both during degradation occurring in oxidative and inert atmosphere, is maintained in a relatively higher and wider temperature range compared to the starting bentonite, as well as to the used organic binding materials. Py-GC/MS studies have shown that at the specified temperatures of 300 °C and 800 °C, only CO_2_ emissions occur from all the analyzed SN/PAA samples. There is no emission of compounds from the BTEX group. This means that the proposed binding material in the form of the MMT-PAA composite will not pose a threat to the environment or workplace.

## Figures and Tables

**Table 1:** Analysis of TG-DSC curves for organobentonites.

Material	Atmosphere	Mass Change Δ*m*, %	Temperature Range, °C	Maximum Rate of Mass Change, °C	DSC Effect Peak, °C	Residual Mass
SN	oxidative	–6.6	30–110	74.5	85.0 (endo)	86.8%
		–2.0	110–170	–	144.0 (endo)	
		−4.6	170–800	665.3	465.9 (exo)	
					663.7 (endo)	
	inert	–1.3	30–110	45.2	142.3 (endo)	92.3%
		–1.4	110–170	135.8	–	
		–5.0	170–800	653.2	–	
					681.0 (endo)	
SN/5PAA	oxidative	–7.0	30–120	56.1	83.9 (endo)	79.2%
		–3.0	120–180	142.4	144.3 (endo)	
		–6.1	180–440	353.6	364.2 (exo)	
		–4.7	440–800	661.7	698.3 (exo)	
	inert	–1.8	30–190	133.7	142.2 (endo)	90.2%
		–1.7	190–360	297.7	–	
		–3.3	360–520	474.1	530.3 (exo)	
		–3.0	520–800	646.7	–	
SN/15PAA	oxidative	–9.1	30–200	71.1	77.5 (endo)	76.0%
		–10.2	200–480	329.8	387.9 (exo)	
		–4.7	480–800	627.1	–	
	inert	–1.6	30–160	112.1	122.1 (endo)	84.3%
		–5.2	160–360	327.5	–	
		–6.3	360–535	436.5	416.3 (exo)	
		–2.6	535–800	616.5	553.8 (exo)	
SN/25PAA	oxidative	–7.2	30–140	49.6	75.4 (endo)	73.1%
		–13.6	140–435	327.5	381.5 (exo)	
		–6.1	435–800	618.6	–	
	inert	–3.2	30–155	92.7	100.0 (endo)	79.7%
		–6.3	155–350	321.1	405.2 (exo)	
		–7.0	350–520	459.1	543.2 (exo)	
		–3.8	520–800	610.1	–	

**Table 2:** Results of Py-GC/MS measurements for PAA and SN/PAA samples at different pyrolysis temperatures.

No.	IUPAC Name	No. CAS	Mass Weight *M*_w_, u	Retention Time *R_T_*, min			
				PAA	SN/5PAA	SN/15PAA	SN/25PAA
1	carbon dioxide	124-38-9	44	2.27 ^a^ 2.29 ^b^	2.20 ^a^ 2.27 ^b^	2.21 ^a^ 2.23 ^b^	2.24 ^a^ 2.25 ^b^
2	benzene	71-43-2	78	4.76 ^a^	-	-	-
3	toluene	108-88-3	92	7.74 ^a^	-	-	-

^a^ Compound detected at 300 °C. ^b^ Compound detected at 800 °C.

## Data Availability

The data are contained within the article and/or available on request from the corresponding author.

## References

[1] Modabberi S., Namayandeh A., Setti M., López-Galindo A. (2019). Genesis of the Eastern Iranian Bentonite Deposits. Appl. Clay Sci..

[2] Guégan R. (2019). Organoclay Applications and Limits in the Environment. C. R. Chim..

[3] Kadir S., Ülah T., Önalgil N., Erkoyun H., Elliott W.C. (2017). Mineralogy, Geochemistry, and Genesis of Bentonites in Miocene Volcanic-Sedimentary Units of the Ankara-Çankiri Basin, Central Anatolia, Turkey. Clays Clay Min..

[4] Lewandowski J.L. (1997). Tworzywa Na Formy Odlewnicze.

[5] Holtzer M., Kmita A., Roczniak A. (2016). Processes of Pyrolysis and Their Effect on Cast Quality and Working Conditions. Pr. Inst. Odlew..

[6] Vasková I., Hrubovčáková M. (2014). Ecological Binding Material of First Generation. Arch. Foundry Eng..

[7] Holtzer M., Grabowska B., Żymankowska-Kumon S., Kwaśniewska-Królikowska D., Dańko R., Solarski W., Bobrowski A. (2012). Harmfulness of Moulding Sands with Bentonite and Lustrous Carbon Carriers. Metalurgija.

[8] Miller L., Vakili R., St-Onge J., Wang Z. (2019). Green Sand Emissions and the Concentration of Carbonaceous Additives. Mod. Cast. Mag..

[9] Kumara P., Bhat V., Banagara A. (2016). Effect of Wood Flours on Moulding Sand Properties. IJRDO J. Mech. Civ. Eng..

[10] Pagacz J., Pielichowski K. (2007). Modyfikacja Krzemianów Warstwowych Do Zastosowań w Nanotechnologii. Czas. Tech. Chem..

[11] Lagaly G., Ogawa M., Dékány I. (2013). Clay Mineral-Organic Interactions. Developments in Clay Science.

[12] He H., Ma L., Zhu J., Frost R.L., Theng B.K.G., Bergaya F. (2014). Synthesis of Organoclays: A Critical Review and Some Unresolved Issues. Appl. Clay Sci..

[13] Cukrowicz S., Sitarz M., Kornaus K., Kaczmarska K., Bobrowski A., Gubernat A., Grabowska B. (2021). Organobentonites Modified with Poly(Acrylic Acid) and Its Sodium Salt for Foundry Applications. Materials.

[14] Huang P., Kazlauciunas A., Menzel R., Lin L. (2017). Determining the Mechanism and Efficiency of Industrial Dye Adsorption through Facile Structural Control of Organo-Montmorillonite Adsorbents. ACS Appl. Mater. Interfaces.

[15] Mousavi M., Fini E.H., Hung A.M. (2019). Underlying Molecular Interactions between Sodium Montmorillonite Clay and Acidic Bitumen. J. Phys. Chem. C.

[16] Shen C.C., Petit S., Li C.J., Li C.S., Khatoon N., Zhou C.H. (2020). Interactions between Smectites and Polyelectrolytes. Appl. Clay Sci..

[17] Cukrowicz S., Goj P., Stoch P., Bobrowski A., Tyliszczak B., Grabowska B. (2021). Molecular Dynamic (MD) Simulations of Organic Modified Montmorillonite. Appl. Sci..

[18] Shirsath S.R., Hage A.P., Zhou M., Sonawane S.H., Ashokkumar M. (2011). Ultrasound Assisted Preparation of Nanoclay Bentonite-FeCo Nanocomposite Hybrid Hydrogel: A Potential Responsive Sorbent for Removal of Organic Pollutant from Water. Desalination.

[19] Meyers K.S., Speyer R.F. (2003). Thermal Analysis of Clays. Handbook of Thermal Analysis and Calorimetry.

[20] Önal M., Sarıkaya Y. (2007). Thermal Behavior of a Bentonite. J. Therm. Anal. Calorim..

[21] Çaykara T., Güven O. (1998). Effect of Preparation Methods on Thermal Properties of Poly(Acrylic Acid)/Silica Composites. J. Appl. Polym. Sci..

[22] Singh B., Sharma N. (2008). Mechanistic Implications of Plastic Degradation. Polym. Degrad. Stab..

[23] Grabowska B. (2013). Nowe Spoiwa Polimerowe w Postaci Wodnych Kompozycji z Udziałem Poli(Kwasu Akrylowego) Lub Jego Soli i Modyfikowanego Biopolimeru do Zastosowania w Odlewnictwie.

[24] Grabowska B., Cukrowicz S., Bobrowski A., Drożyński D., Żymankowska-Kumon S., Kaczmarska K., Tyliszczak B., Pribulová A. (2023). Organobentonite Binder for Binding Sand Grains in Foundry Moulding Sands. Materials.

[25] Bayram H., Önal M., Yılmaz H., Sarıkaya Y. (2010). Thermal Analysis of a White Calcium Bentonite. J. Therm. Anal. Calorim..

[26] Kamińska J., Puzio S., Angrecki M. (2020). Effect of Bentonite Clay Addition on the Thermal and Mechanical Properties of Conventional Moulding Sands. Arch. Foundry Eng..

[27] Paź S., Drożyński D., Górny M., Cukrowicz S. (2019). Properties of Bentonites and Bentonite Mixtures Used in Casting Processes. Arch. Foundry Eng..

[28] Alves J.L., e Rosa P.d.T.V., Realinho V., Antunes M., Velasco J.I., Morales A.R. (2020). The Effect of Brazilian Organic-Modified Montmorillonites on the Thermal Stability and Fire Performance of Organoclay-Filled PLA Nanocomposites. Appl. Clay Sci..

[29] Grabowska B., Sitarz M., Olejnik E., Kaczmarska K. (2015). FT-IR and FT-Raman Studies of Cross-Linking Processes with Ca^2+^ Ions, Glutaraldehyde and Microwave Radiation for Polymer Composition of Poly(Acrylic Acid)/Sodium Salt of Carboxymethyl Starch-Part I. Spectrochim. Acta A Mol. Biomol. Spectrosc..

[30] Grabowska B., Sitarz M., Olejnik E., Kaczmarska K., Tyliszczak B. (2015). FT-IR and FT-Raman Studies of Cross-Linking Processes with Ca^2+^ Ions, Glutaraldehyde and Microwave Radiation for Polymer Composition of Poly(Acrylic Acid)/Sodium Salt of Carboxymethyl Starch—In Moulding Sands, Part II. Spectrochim. Acta A Mol Biomol. Spectrosc..

[31] Grabowska B., Hodor K., Kaczmarska K., Bobrowski A., Kurleto-Kozioł Ż., Fischer C. (2017). Thermal Analysis in Foundry Technology: Part 2. TG–DTG–DSC, TG–MS and TG–IR Study of the New Class of Polymer Binders BioCo. J. Therm. Anal. Calorim..

[32] Grabowska B., Żymankowska-Kumon S., Cukrowicz S., Kaczmarska K., Bobrowski A., Tyliszczak B. (2019). Thermoanalytical Tests (TG–DTG–DSC, Py-GC/MS) of Foundry Binders on the Example of Polymer Composition of Poly(Acrylic Acid)–Sodium Carboxymethylcellulose. J. Therm. Anal. Calorim..

[33] Yang Z., Peng H., Wang W., Liu T. (2010). Crystallization behavior of poly(ε-caprolactone)/layered double hydroxide nanocomposites. J. Appl. Polym. Sci..

[34] Svidró J.T., Diószegi A., Svidró J., Ferenczi T. (2017). The Effect of Different Binder Levels on the Heat Absorption Capacity of Moulding Mixtures Made by the Phenolic Urethane Cold-Box Process. J. Therm. Anal. Calorim..

[35] Svidró J.T., Diószegi A., Svidró J., Ferenczi T. (2017). Thermophysical Aspects of Reclaimed Moulding Sand Addition to the Epoxy-SO2 Coremaking System Studied by Fourier Thermal Analysis. J. Therm. Anal. Calorim..

[36] Ikhtiyarova G.A., Özcan A.S., Gök Ö., Özcan A. (2012). Characterization of Natural- and Organobentonite by XRD, SEM, FT-IR and Thermal Analysis Techniques and Its Adsorption Behaviour in Aqueous Solutions. Clay Miner..

[37] Leite I.F., Soares A.P.S., Carvalho L.H., Raposo C.M.O., Malta O.M.L., Silva S.M.L. (2010). Characterization of Pristine and Purified Organobentonites. J. Therm. Anal. Calorim..

[38] Zhu Y., Cui Y., Shan Z., Dai R., Shi L., Chen H. (2021). Fabrication and Characterization of a Multi-Functional and Environmentally-Friendly Starch/Organo-Bentonite Composite Liquid Dust Suppressant. Powder Technol..

